# Efficacy and safety analysis of non-radical surgery for early-stage cervical cancer (IA2 ~ IB1): a systematic review and meta-analysis

**DOI:** 10.3389/fmed.2024.1337752

**Published:** 2024-04-30

**Authors:** Siyuan Zeng, Simin Xiao, Yang Xu, Ping Yang, Chenming Hu, Xianyu Jin, Lifeng Liu

**Affiliations:** ^1^Department of Obstetrics and Gynecology, Dalian Municipal Central Hospital, Dalian, China; ^2^Dalian Municipal Central Hospital, China Medical University, Shenyang, China; ^3^Radiology Department, XinDu Hospital of Traditional Chinese Medicine, Chengdu, China; ^4^Department of Radiation Oncology, The First Affiliated Hospital of Dalian Medical University, Dalian, China; ^5^School of Clinical Medicine, North Sichuan Medical College, Nanchong, China

**Keywords:** early stage, cervical cancer, conservative surgery, less radical surgery, simple hysterectomy

## Abstract

**Objective:**

Radical hysterectomy has long been considered as the standard surgical treatment for early-stage cervical cancer (IA2 to IB1 stages), according to the 2009 International Federation of Obstetrics and Gynecology. This study aims to conduct an in-depth evaluation of the effectiveness and safety of non-radical surgery as an alternative treatment for patients with early-stage cervical cancer.

**Methods:**

A systematic search of online databases including PubMed, Embase, and the Cochrane Library was conducted to identify relevant literature on surgical treatment options for early-stage cervical cancer. Keywords such as “cervical cancer,” “conservative surgery,” “early-stage,” “less radical surgery,” and “simple hysterectomy” were used. Meta-analysis was performed using Stata 15.0 software, which included randomized controlled trials (RCTs) and cohort studies.

**Results:**

This meta-analysis included 8 eligible articles covering 9 studies, with 3,950 patients in the simple hysterectomy (SH) surgery group and 6,271 patients in the radical hysterectomy (RH) surgery group. The results indicate that there was no significant difference between the two groups in terms of the Overall Survival (OS) (HR = 1.04, 95% CI: 0.86–1.27, *p* = 0.671; Heterogeneity: *I*^2^ = 33.8%, *p* = 0.170), Disease Free Survival (DFS) (HR = 1.39, 95% CI: 0.59–3.29, *p* = 0.456; Heterogeneity: *I*^2^ = 0.0%, *p* = 0.374), Cervical Cancer Specific Survival (CCSS) (HR = 1.11, 95% CI: 0.80–1.54, *p* = 0.519; Heterogeneity: *I*^2^ = 11.9%, *p* = 0.287) and recurrence rate (RR = 1.16, 95% CI: 0.69–1.97, *p* = 0.583; Heterogeneity: *I* = 0.0%, *p* = 0.488). However, the mortality rate (RR = 1.35, 95% CI: 1.10–1.67, *p* = 0.006; Heterogeneity: *I*^2^ = 35.4%, *p* = 0.158) and the rate of postoperative adjuvant therapy (RR = 1.59, 95% CI: 1.16–2.19, *p* = 0.004; Heterogeneity: *I*^2^ = 92.7%, *p* < 0.10) were higher in the SH group compared to those in the RH group. On the other hand, the incidence of surgical complications was lower in the SH group (RR = 0.36, 95% CI: 0.21–0.59, *p* = 0.004; Heterogeneity: *I*^2^ = 0.0%, *p* = 0.857) than that in the RH group. Subgroup analysis revealed that patients in the IB1 stage SH group had a significantly higher mortality rate compared to those in the RH group (RR = 1.59, 95% CI: 1.23–2.07, *p* < 0.001; Heterogeneity: *I*^2^ = 0.0%, *p* = 0.332). However, there was no significant difference in mortality rates between the two groups for patients at stage IA2 (RR = 0.84, 95% CI: 0.54–1.30, *p* = 0.428; Heterogeneity: *I*^2^ = 26.8%, *p* = 0.243). In the subgroups positive for Lymphovascular Space Invasion (LVSI), patients in the SH group had a significantly higher mortality rate than those in the RH group (RR = 1.34, 95% CI: 1.09–1.65, *p* = 0.005; Heterogeneity: *I*^2^ = 41.6%, *p* = 0.128). However, in the LVSI-negative subgroups, there was no significant difference in mortality rates between the two groups (RR = 0.33, 95% CI: 0.01–8.04, *p* = 0.499).

**Conclusion:**

For patients with early-stage cervical cancer patients at IA2 without LVSI involvement, comparisons between the two groups in terms of OS, DFS, CCSS, recurrence rate, and mortality rates revealed no statistically significant differences, indicating that the choice of surgical approach does not affect long-term survival outcomes for this specific patient group. For patients at IB1 and IA2 stages with LVSI involvement, while there were no significant differences between the two groups in OS, DFS, CSS, and recurrence rate, a significant increase in mortality rates was observed in the SH group. This indicates a potential elevated risk of mortality associated with SH in this subset of patients. Notably, the incidence of surgical complications was significantly lower in the SH group compared to the RH group, highlighting the safety profile of SH in this context. Significantly, among patients in the SH group, an increase in the rate of postoperative adjuvant treatment is associated with a higher occurrence of treatment-related complications. To facilitate more precise patient selection for conservative surgical management, future prospective studies of superior quality are imperative to gain deeper insights into this matter.

**Systematic review registration:**

PROSPERO (CRD42023451609: https://www.crd.york.ac.uk/prospero/display_record.php?ID=CRD42023451609).

## 1 Introduction

Presently, cervical cancer stands as a prevalent malignancy within the female reproductive tract, securing its position as the fourth most threatening cancer to women’s health, following breast, colorectal, and lung cancers. It has become a significant global public health issue. In 2020, there were 604,000 new cases of cervical cancer worldwide, with an incidence rate of 15.6 per 100,000 people, and 342,000 deaths, resulting in a mortality rate of 8.8 per 100,000 people (1). Fortunately, thanks to the widespread availability of screening technologies, an increasing number of cervical cancer cases are being diagnosed at an early stage.

For patients with cervical cancer at International Federation of Gynecology and Obstetrics (FIGO) stages IA2 to IB1, Radical Hysterectomy (RH) combined with pelvic lymphadenectomy is considered the fundamental approach for early-stage cervical cancer treatment (2). However, the traditional RH procedure involves the removal of the uterine main ligaments, sacrouterine ligaments, and parametrial tissue, a process that may inflict damage on the pelvic autonomic nervous system, leading to disruption of the pelvic floor support structure’s anatomy (3, 4). These factors increase the risk of perioperative complications such as bleeding, damage to the ureter and bladder, and postoperative complications like fistulas, urinary retention or incontinence, and sexual dysfunction (5). Although past National Comprehensive Cancer Network (NCCN) guidelines have recommended radical hysterectomy for patients with IA2 and IB1 stage cervical cancer, there is currently no RCT evidence to prove that this standard surgical procedure, which has a history of over 120 years, has better oncological outcomes compared to non-radical surgeries (6).

To further investigate the feasibility of employing non-radical surgical approaches for patients with early-stage cervical cancer, this study utilizes a meta-analysis method to synthesize findings from relevant clinical research. We compared the effectiveness and safety of SH versus RH in the treatment of early-stage cervical cancer, aiming to assess the relative merits of these two surgical techniques.

## 2 Methods

The meta-analysis adhered to the Preferred Reporting Items for Systematic Reviews and Meta-Analyses (PRISMA) guidelines (7).

### 2.1 Data selection

#### 2.1.1 Research types

Randomized controlled trials (RCTs), observational studies, cohort studies, etc.

#### 2.1.2 Research subjects

The study encompassed women diagnosed with early-stage cervical cancer, specifically stages IA2 to IB1. Research articles that merged data from IA2 and IB1 stages with additional stages (including IA1 LVSI, IB2, and IIA) were incorporated into the table and appropriately annotated. The analysis covered histological variants like adenocarcinoma, squamous cell carcinoma, and adenosquamous carcinoma.

#### 2.1.3 Intervention measures

The control group was treated with a RH ± Pelvic lymphadenectomy, while the experimental group received SH treatment ± Pelvic lymphadenectomy.

### 2.2 Outcome measures

Primary outcome measure: Overall Survival (OS) refers to the duration from the moment of randomization to death from any cause. Secondary Outcome Measures include: 1. Disease-Free Survival (DFS): Calculated from the initiation of treatment, it represents the period throughout which a patient remains free from any recurrence or progression of cervical cancer. 2. Cervical Cancer-Specific Survival (CCSS): CCSS measures the time from treatment initiation to death specifically caused by cervical cancer. 3. Mortality Rate: This encompasses the proportion of patients who pass away due to any cause following cervical cancer treatment, during the follow-up period. 4. Recurrence Rate: Referring to the proportion of cervical cancer patients experiencing disease reappearance after treatment during the follow-up period, recurrence is strictly defined as an invasive event, excluding any *in situ* developments. 5. Postoperative Adjuvant Therapy Rate: This measure indicates the proportion of patients that receive additional adjuvant treatment (e.g., chemotherapy, radiotherapy, or hormone therapy) subsequent to surgical intervention. 6. Incidence of Surgical Complications: This quantifies the frequency at which patients encounter complications during and after surgery.

### 2.3 Literature screening and data extraction

A thorough systematic literature search was conducted to identify relevant studies investigating the outcomes of SH in women with early-stage cervical cancer (IA2 to IB1). A comprehensive search strategy employing keywords including “early-stage cervical cancer,” “simple hysterectomy,” and “radical hysterectomy” was implemented across multiple databases such as Embase, PubMed, Cochrane Library, and other relevant sources. In addition, a manual screening of references was performed to ensure maximum coverage of the available literature. Studies meeting the following exclusion criteria were excluded from the analysis: (1) duplicate publications; (2) studies lacking essential data for the present research; (3) studies published in languages other than English; (4) overview articles, case reports, conference papers, and similar sources; (5) studies focusing on histological types other than those specified, such as clear cells, serous, and neuroendocrine; (6) studies reporting new trials of adjuvant therapies, including chemotherapy (CT) or radiotherapy; and (7) studies solely examining IA1 or lower or IB2 or higher stages, unless these results were combined with IA2 to IB1 cases. Data extraction from each included study was performed using standardized tables, capturing relevant information such as the first author, publication year, study design, study population, intervention measures, outcome indicators, and more. Notably, our research has been prospectively registered with the PROSPERO database (registration number: CRD42023451609), ensuring transparency and accountability in the research process.

### 2.4 Literature quality evaluation

To evaluate the quality of the retrospective survey, a 9-star Newcastle Ottawa Scale (NOS) was used, with the lowest 6 stars being considered as high quality (8). The RCTs included in the study referred to the Cochrane Collaboration Network’s setting of the bias risk assessment entry (9), and the bias risk of each study was independently evaluated: A random number table, computer randomization, coin tossing, poker or envelope washing, drawing lots, and rolling dice with a score of 1 point were used. Additionally, the blind method was used to give 1 point, 1 point was for not losing outcome data, and propensity analysis was used, 1 point was for reporting non-selective outcomes, The study showed no other sources of bias, giving a score of 1, The total score was calculated based on each score. Finally, calculate the total score for each study based on these criteria.

### 2.5 Statistical analysis methods

The meta-analyses were conducted using Stata 15.0 software. Initially, the heterogeneity among included studies was assessed using the Chi-square test (test level *α* = 0.10). If no statistical heterogeneity was detected among the studies (*p* > 0.05, *I*^2^ < 50%), a fixed-effect model was employed for analysis; conversely, if statistical heterogeneity was present (*p* < 0.10, *I*^2^ > 50%), a random-effects model was utilized, with subgroup analyses and sensitivity analyses conducted to explore the sources of heterogeneity. Hazard Ratios (HR) and 95% Confidence Intervals (CI) were employed to evaluate OS, DFS, and CCSS, while Relative Risks (RR) and 95% CI were utilized to assess mortality rates, recurrence rates, rates of postoperative adjuvant therapy, and incidence of surgical complications. Publication bias was evaluated through Egger’s test, Begg’s test, and funnel plots with Stata 15.0. Subgroup analyses, for instance, based on study type, disease staging, sample size, and other factors, were planned within the meta-analysis for detailed examination and comparison. Furthermore, sensitivity analyses were conducted to assess the impact of the quality of included studies on the overall findings. Results from the meta-analysis, including effect sizes and confidence intervals, were graphically represented using forest plots and other methods as necessary. Additional statistical techniques were applied as needed to interpret and analyze heterogeneity among studies. All findings were presented in tables and graphs, accompanied by concise concluding paragraphs.

## 3 Results

### 3.1 Process diagram of literature retrieval

**Figure 1:**
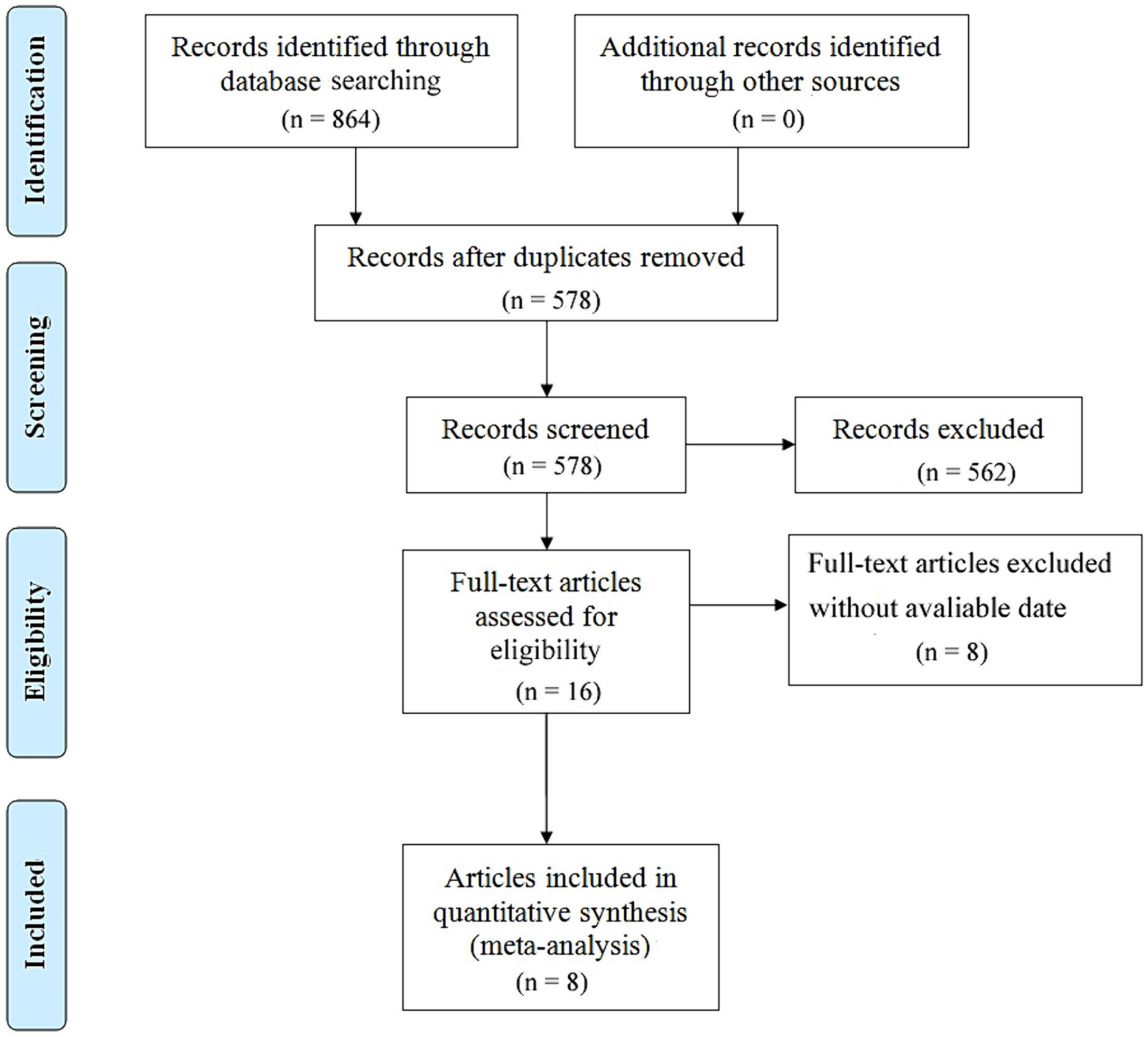
Flow chart of the meta-analysis.

After conducting a comprehensive search across major databases, a total of 864 relevant articles were identified. Following the removal of duplicates, 578 articles remained. Subsequently, a detailed review of the titles and abstracts of these articles was carried out based on predefined inclusion and exclusion criteria, resulting in 562 articles being excluded. After a thorough full-text review, 8 articles covering 9 studies were ultimately selected, involving literature numbered (10–17). The specific selection process is illustrated in Figure 1.

### 3.2 Basic characteristics of collected literature

This study included a total of 10,221 early-stage cervical cancer patients, comprising 3,950 patients who underwent SH (Querleu-Morrow type A and Piver type I hysterectomy) and 6,271 patients who received RH (Querleu-Morrow type B (B1 + B2) and type C (C1 + C2), Piver Type II and type III radical hysterectomy). In these 9 studies, all patients were diagnosed with cervical cancer ranging from stages IA2 to IIA. Among the studies, six explicitly reported using the 2009 version of the FIGO staging system, while the other three did not specify which version of the FIGO staging was used. However, all nine studies provided detailed reports on the maximum diameter of the tumor and information that is crucial for understanding and assessing the clinical relevance of the study findings. The types of studies encompassed 4 RCTs and 4 cohort studies. Of these, 4 studies were conducted in the United States, 3 in China, and 2 in other countries, with sample sizes ranging from 40 to 3,931. Table 1 provides the basic information of the original literature. Table 2 lists the patients’ characteristics from the original literature. The pathological types of tumors among the included cervical cancer patients consisted of adenocarcinoma, squamous cell carcinoma, and adenosquamous carcinoma. The vast majority of patients (87.53%) had tumors with a diameter of less than 2 cm, and the tumor diameter in all patients was less than 4 cm. Approximately 14.44% of cases were positive for LVSI. Among all patients who underwent lymph node (LN) assessment, about 5.75% demonstrated positive LN.

**Table 1:** Characteristics of studies included in this meta-analysis.

Study, year	Study design	Country	Duration	Sample size (SH/RH)	Age (SH/RH)	Median follow-up (months)	Stage (year)	Summary statistics
Landoni 2012	RCT	Italy	1981–1986	62/63	55 (34–82)/44 (24–72)	≥280	IB1-IIA (NR)	OS: HR: 0.53 (0.25–1.12) DFS: 70% SH, 86% RH
Wang 2017	RCT	China	2002–2014	70/70	44.04 ± 8.46/43.03 ± 8.59	75	IB1 (2009)	OS: 100% SH, 98.5% RH (*p* = 0.32) RFS: HR 0.49 (0.04–5.37)
Chen 2018	RCT	China	2006–2011	45/56	50 (34–75)/47 (24–72)	≥60	IA2-IB1 (2009)	OS: HR: 0.49 (0.12–2.10)
Tseng 2018	Cohort	USA	1998–2012	807/1764	median: 37	79	IB1 (2009)	DSS: HR: 1.01 (0.69–1.45)
Sia 2019 1	Cohort	USA	2004–2015	683/847	NR	56	IA2 (NR)	OS: HR: 0.68 (0.37–1.25)
Sia 2019 2	Cohort	USA	2004–2015	1388/2543	NR	53	IB1 (NR)	OS: HR: 1.31 (0.97–1.75)
Liu 2021	Cohort	China	2014–2019	182/258	44.5 ± 12.8/44.3 ± 12.3	39/45	IA2 (2009)	OS: HR: 1.122 (0.319–3.493) DFS: HR: 1.608 (0.640–4.041)
Du 2022	Cohort	USA	1998–2015	693/650	NR	97/107	IA2 (2009)	OS: HR: 1.078 (0.764–1.522) CSS: HR: 1.536 (0.782–3.021)
Carneiro 2023	RCT	Brazil	2015–2018	20/20	37 (34–50.5)/37.5 (34–44)	52.1	IA2-IB1 (2009)	OS: HR: 0.48 (0.07–3.35) DFS: 95% SH, 100% RH (*p* = 0.30)

SH, simple hysterectomy; RH, radical hysterectomy; OS, overall survival; DFS, disease-free survival; NR, not reached.

**Table 2:** Patients characteristics of studies included in this meta-analysis.

Study, year	Type of surgery		Tumor size		LVSI(+)	LN(+)	Adjuvant therapy		Recurrences		Deaths		Complications	
	RH	SH	<2 cm	2–4 cm			RH	SH	RH	SH	RH	SH	RH	SH
Landoni 2012	63	62	8	117	52	13	35	43	8	14	12	18	24	8
Wang 2017	70	70	140	0	0	4	2	2	2	1	1	0	10	3
Chen 2018	56	45	101	0	25	0	23	22	10	5	5	3	12	3
Tseng 2018	1764	807	1,414	1,157	NR	444	507	217	NR	NR	NR	NR	NR	NR
Sia 2019 1	847	683	1,530	0	143	17	79	138	NR	NR	38	22	NR	NR
Sia 2019 2	2,543	1,388	3,931	0	615	48	496	578	NR	NR	111	98	NR	NR
Liu 2021	258	182	440	0	67	NR	23	48	6	5	11	10	NR	NR
Du 2022	650	693	1,343	0	NR	34	87	150	NR	NR	NR	NR	NR	NR
Carneiro 2023	20	20	40	0	9	3	4	6	0	1	1	2	5	3
Total	6,271 (61.3%)	3,950 (38.6%)	8,947 (87.5%)	1,274 (12.4%)	911 (14.4%)	563 (5.7%)	1,256 (20%)	1,204 (30.4%)	26 (3%)	26 (3%)	179 (4.6%)	153 (6.2%)	51 (24.4%)	17 (8.6%)

SH, simple hysterectomy; RH, radical hysterectomy; NR, not reached.

### 3.3 Quality evaluation results of collected literature

In this study, we employed RevMan 5.4 software to assess the risk of bias in RCTs. Among the included four studies, there was a low to moderate risk of bias demonstrated in aspects such as the generation of random sequences, blinding measures implemented for participants and researchers, and the completeness of reported outcomes. Overall, the quality assessment of these RCTs indicates that the studies involved have a moderate risk of bias (Supplementary Figures S1, S2 for details). Furthermore, based on the NOS scores, all five cohort studies included in this meta-analysis were rated as high-quality research (Supplementary Table S1).

### 3.4 Meta-analysis results

#### 3.4.1 OS

**Figure 2:**
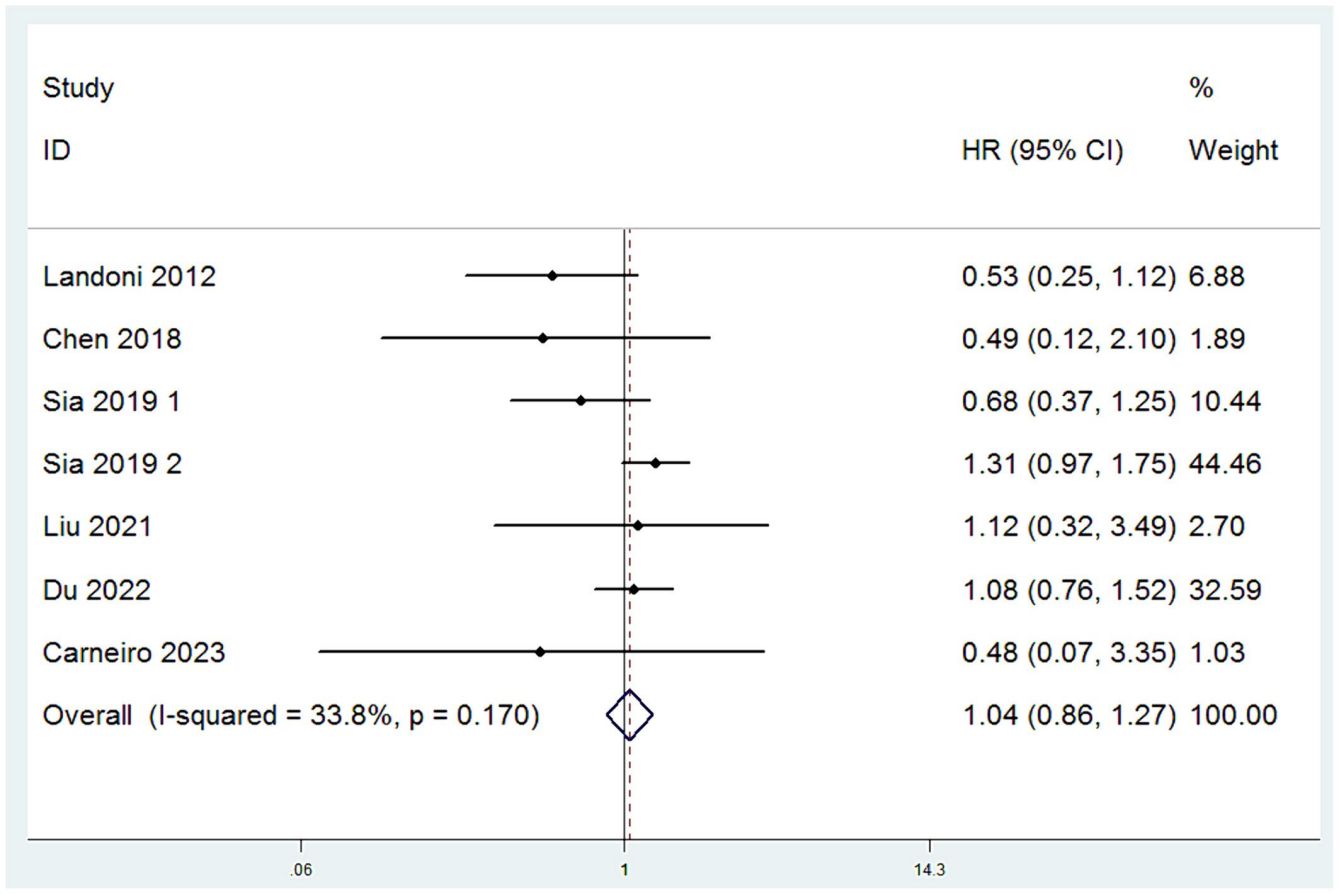
Forest plots for overall survival (OS).

Data on OS were obtained from a comprehensive analysis of seven studies (12–17). The studies exhibited minimal heterogeneity, indicated by an *I*^2^ value less than 50%, which justifies the adoption of a fixed-effect model. (*I*^2^ < 50%) HR served as the effect measure, and the meta-analysis results revealed no significant difference in OS between the SH group and the RH group. Specifically, there was no statistically significant difference in the risk of death between patients in the SH group and those in the RH group (HR = 1.04, 95% CI: 0.86–1.27, *p* = 0.671; Heterogeneity: *I*^2^ = 33.8%, *p* = 0.170) (Figure 2).

#### 3.4.2 DFS

**Figure 3:**
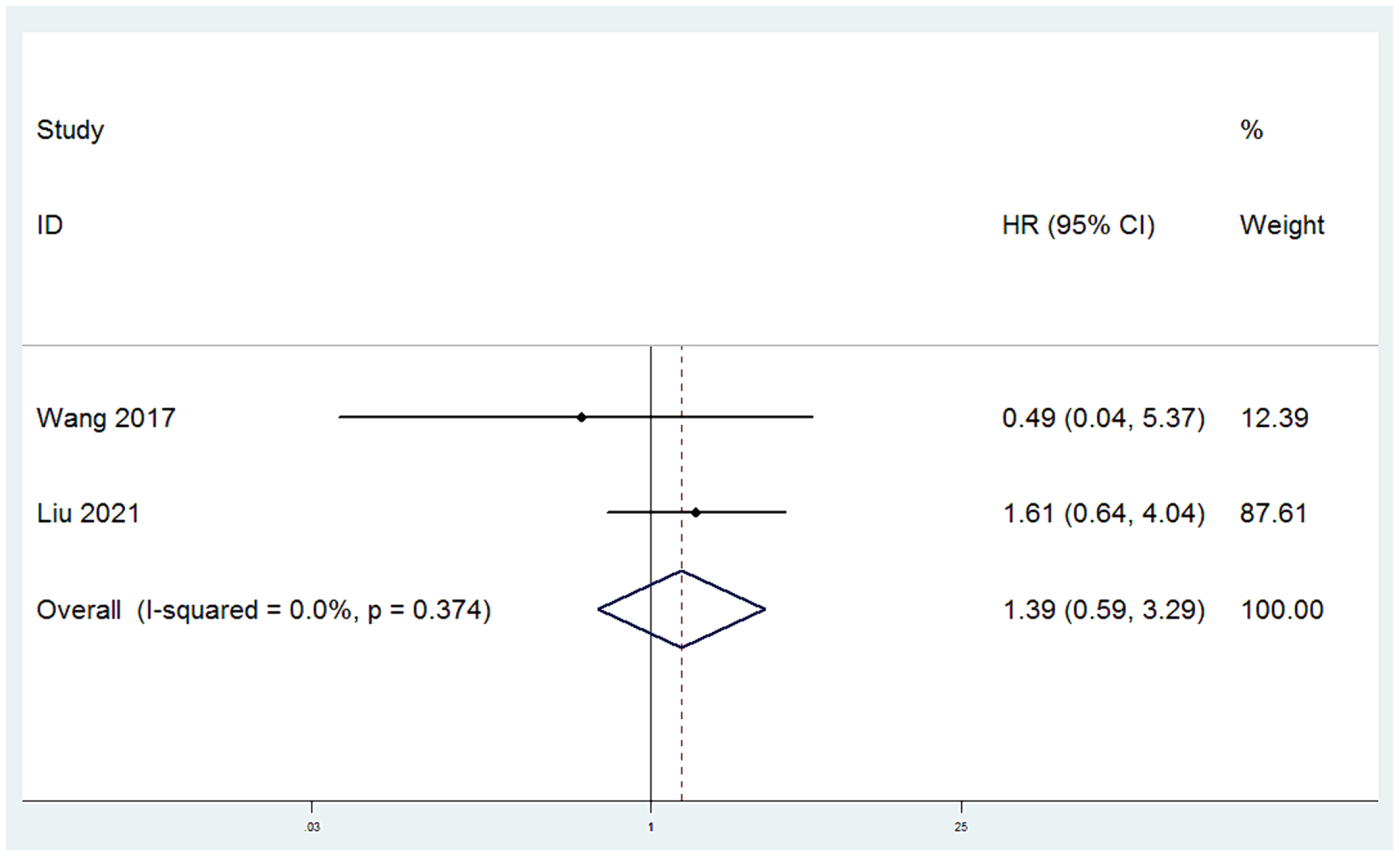
Forest plots for disease free survival (DFS).

DFS were obtained from two studies (10, 13). With low heterogeneity observed between these studies (*I*^2^ < 50%), a fixed-effect model was applied. Utilizing HR as the effect measure, the meta-analysis results indicated that there was no statistically significant difference in DFS between patients in the SH group and those in the RH group, suggesting that the risk of disease recurrence or death was similar for both groups (HR = 1.39, 95% CI: 0.59–3.29, *p* = 0.456; Heterogeneity: *I*^2^ = 0.0%, *p* = 0.374) (Figure 3).

#### 3.4.3 CCSS

**Figure 4:**
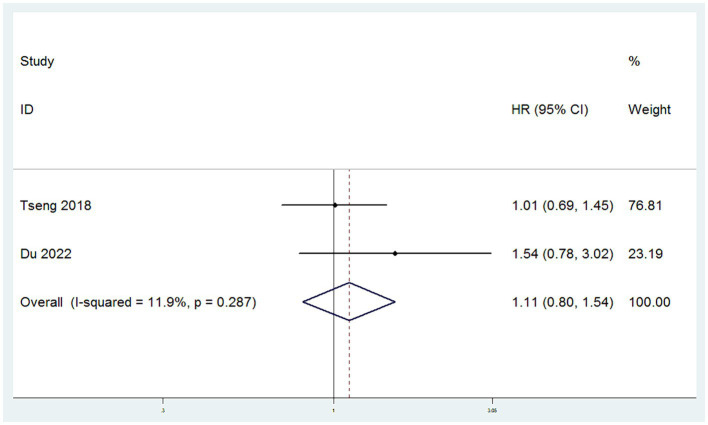
Forest plots for Cervical Cancer Specific Survival Rate/Disease Specific Survival Rate (CCSS).

CCSS were sourced from two studies (11, 15). The heterogeneity among these studies for CCSS was low (*I*^2^ < 50%), leading to the application of a fixed-effect model. Meta-analysis, utilizing HR as the measure of effect, demonstrated that there was no significant difference in CCSS between patients in the SH group and those in the RH group (HR = 1.11, 95% CI: 0.80–1.54, *p* = 0.519; Heterogeneity: *I*^2^ = 11.9%, *p* = 0.287) (Figure 4).

#### 3.4.4 Recurrence rates

**Figure 5:**
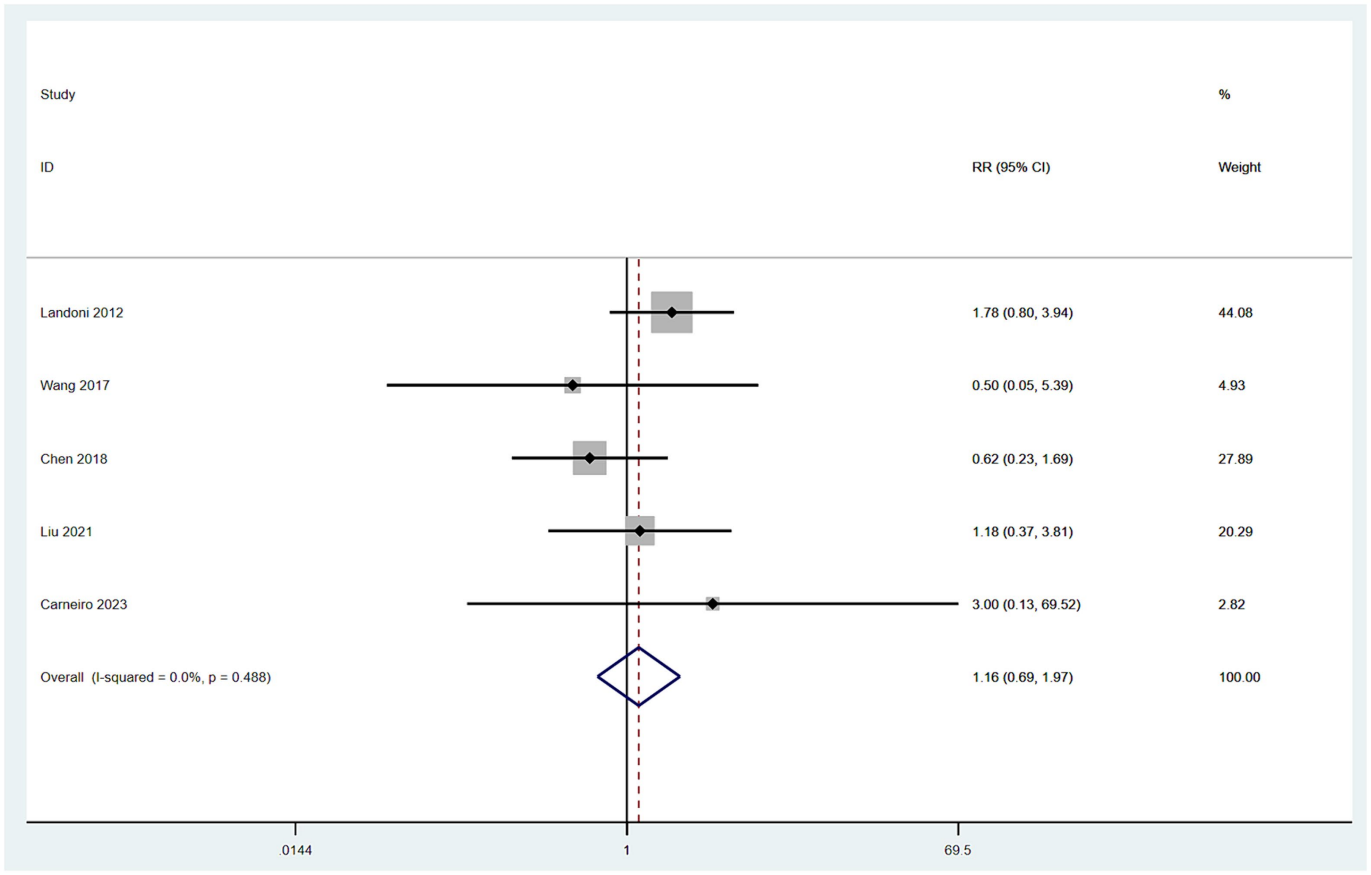
Forest plots for recurrence rate.

Data on recurrence rates were derived from five studies (10, 12–14, 16). With low heterogeneity observed among these studies (*I*^2^ < 50%), a fixed-effect model was employed. The meta-analysis, using RR as the effect measure, found no significant difference in recurrence rates between patients in the SH group and those in the RH group (RR = 1.16, 95% CI: 0.69–1.97, *p* = 0.583; Heterogeneity: *I*^2^ = 0.0%, *p* = 0.488) (Figure 5).

#### 3.4.5 Mortality rates

**Figure 6:**
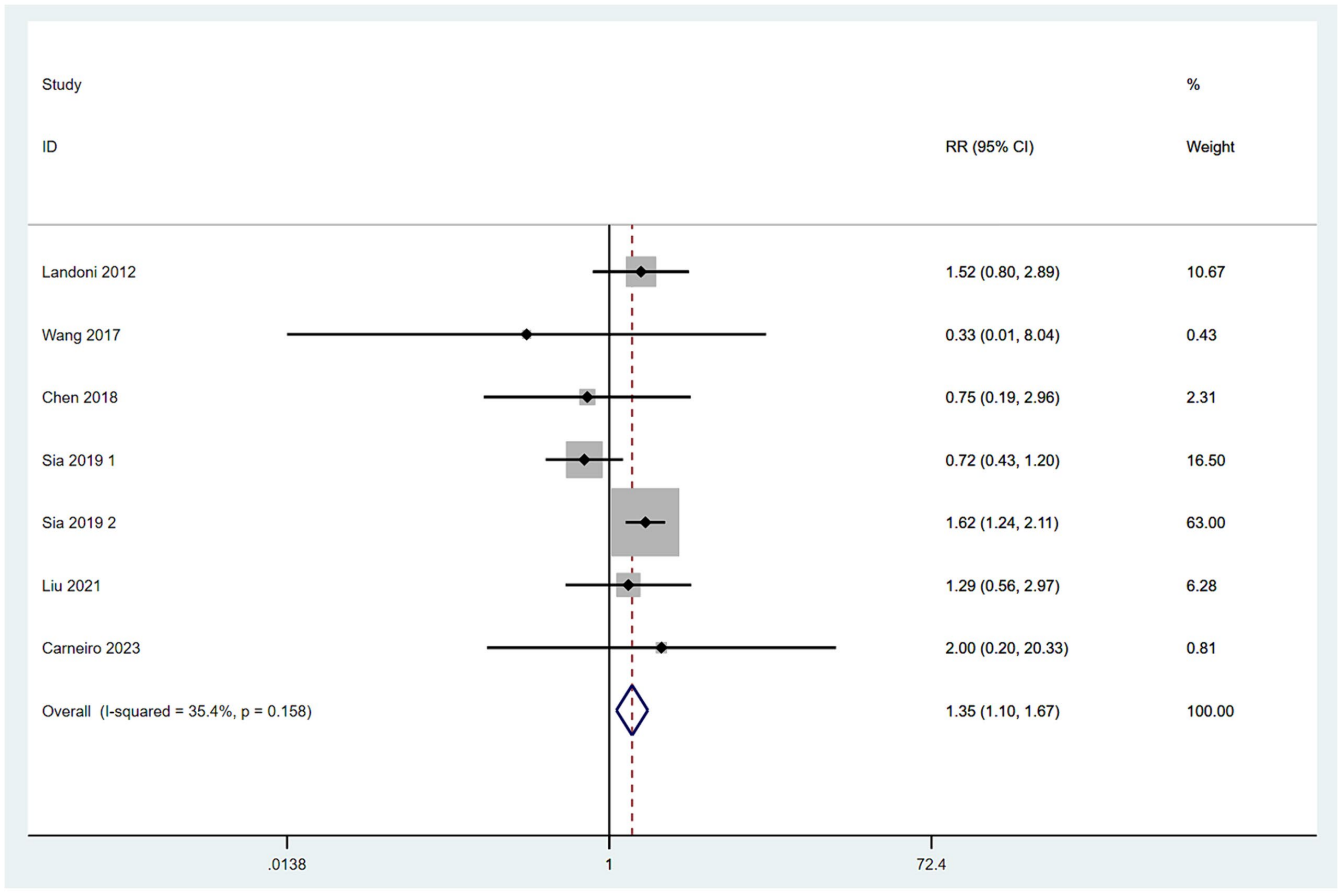
Forest plots for mortality rate.

Data on mortality rates were obtained from seven studies (10–14, 16, 17). With low heterogeneity among these studies (*I*^2^ < 50%), a fixed-effect model was applied. The meta-analysis, utilizing RR as the effect measure, indicated that the mortality rate in the SH group was higher than in the RH group (RR = 1.35, 95% CI: 1.10–1.67, *p* = 0.006; Heterogeneity: *I*^2^ = 35.4%, *p* = 0.158) (Figure 6).

#### 3.4.6 The rate of postoperative adjuvant therapy

**Figure 7:**
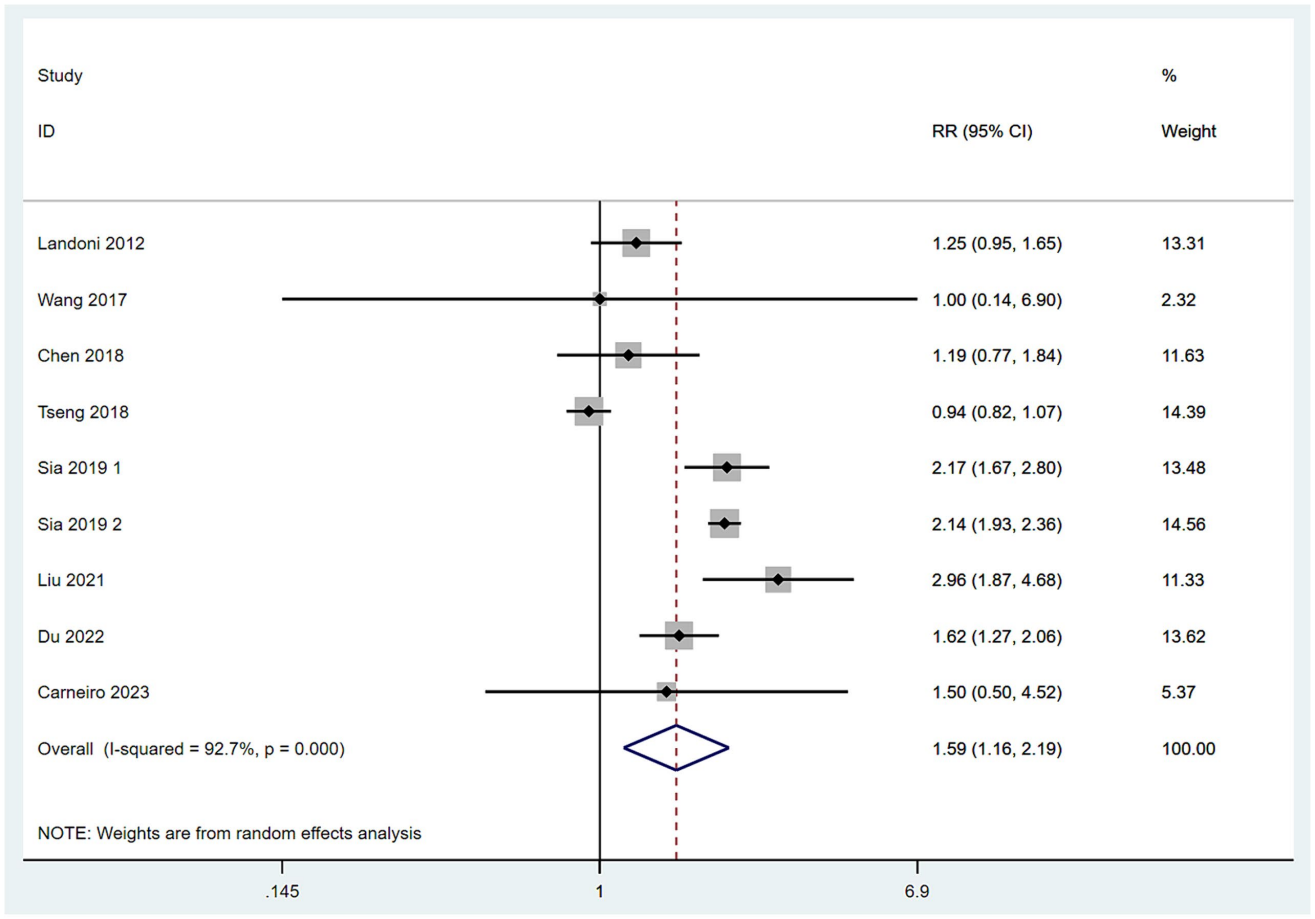
Forest plots for postoperative adjuvant therapy rate.

Data on the rate of postoperative adjuvant therapy were collected from nine studies (10–17). With high heterogeneity among these studies (*I*^2^ > 50%), a random-effects model was employed. The meta-analysis, using RR as the measure of effect, indicated that the rate of postoperative adjuvant therapy in the SH group was higher than in the RH group (RR = 1.59, 95% CI: 1.16–2.19, *p* = 0.004; Heterogeneity: *I*^2^ = 92.7%, *p* < 0.10) (Figure 7).

#### 3.4.7 The incidence of surgical complication

**Figure 8:**
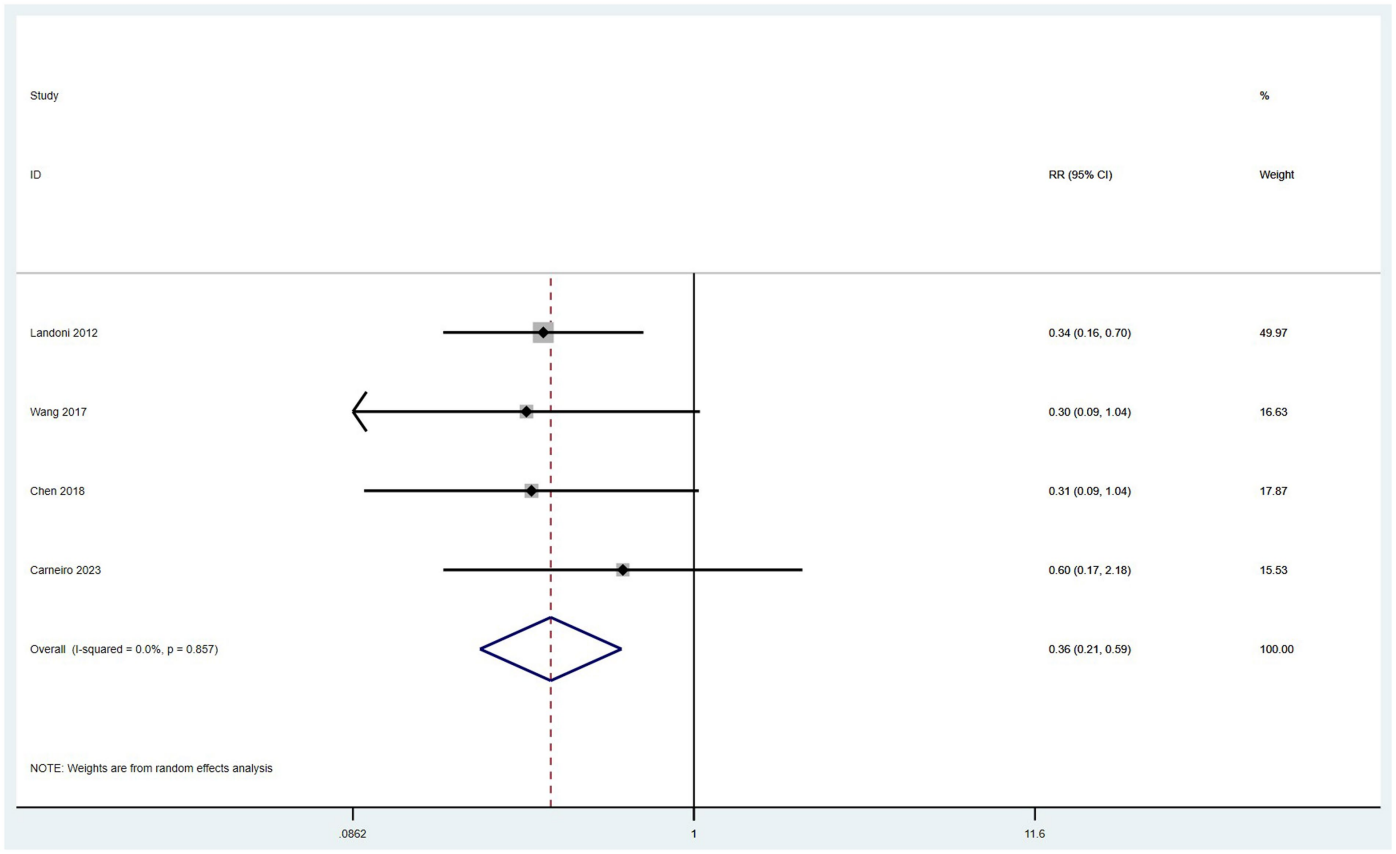
Forest plots for surgical complication rate.

Data on the incidence of surgical complications were derived from four studies (10, 14, 16, 17). With low heterogeneity observed among these studies (*I*^2^ < 50%), a fixed-effect model was applied. The meta-analysis, utilizing RR as the measure of effect, indicated that the incidence of postoperative complications in the SH group was lower than in the RH group (RR = 0.36, 95% CI: 0.21–0.59, *p* < 0.001; Heterogeneity: *I*^2^ = 0.0%, *p* = 0.857) (Figure 8).

### 3.5 Subgroup analysis

An extensive analysis of subgroups based on various influencing factors was conducted and the outcomes are summarized in Table 3. The findings revealed a significant disparity in mortality rates between patients with Stage IB1 cervical cancer who underwent SH compared to those who underwent RH, with the former group exhibiting a notably higher mortality rate (RR = 1.59, 95% CI: 1.23–2.07, *p* < 0.001; heterogeneity: *I*^2^ = 0.0%, *p* = 0.332). Conversely, for patients in the Stage IA2 subgroup, there was no statistically significant variance in mortality rates observed between those who underwent SH and RH procedures (RR = 0.84, 95% CI: 0.54–1.30, *p* = 0.428; heterogeneity: *I*^2^ = 26.8%, *p* = 0.243). Subsequently, within the subgroup of patients testing positive for LVSI, individuals who underwent SH exhibited a significantly higher mortality rate compared to their counterparts who had RH (RR = 1.34, 95% CI: 1.09–1.65, *p* = 0.005; heterogeneity: *I*^2^ = 41.6%, *p* = 0.128). However, there was no statistically significant difference in mortality rates between the SH and RH groups among the LVSI negative subgroup (RR = 0.33, 95% CI: 0.01–8.04, *p* = 0.499). In the LN-positive subgroup, there was no statistically significant difference in mortality rates between the SH and RH groups (RR = 1.23, 95% CI: 0.77–1.94, *p* = 0.384; heterogeneity: *I*^2^ = 53.2%, *p* = 0.073), and this was also true for the LN-negative subgroup (RR = 0.75, 95% CI: 0.19–2.96, *p* = 0.677). Likewise, when stratified by study type, the comparison of mortality rates between the SH and RH groups within RCTs or cohort studies did not yield statistically significant differences. (RR = 1.32, 95% CI: 0.76–2.29, *p* = 0.332; heterogeneity: *I*^2^ = 0.0%, *p* = 0.635) or the cohort study subgroup (RR = 1.16, 95% CI: 0.66–2.05, *p* = 0.599; heterogeneity: *I*^2^ = 73.7%, *p* = 0.022).

**Table 3:** Subgroups analysis for mortality rate.

Subgroup	No. of studies	RR(95%CI)	*p*	*I*^2^ (%)	Ph
Stage					
IA2	2	0.84 (0.54–1.30)	0.428	26.8	0.243
IB1	2	1.59 (1.23–2.07)	<0.001	0	0.332
Study design					
RCTs	4	1.32 (0.76–2.29)	0.332	0	0.635
Cohort	3	1.16 (0.66–2.05)	0.599	73.7	0.022
LVSI					
Positive	6	1.34 (1.09–1.65)	0.005	41.6	0.128
Negative	1	0.33 (0.01–8.04)	0.499	–	–
LN					
Positive	5	1.23 (0.77–1.94)	0.384	53.2	0.073
Negative	1	0.75 (0.19–2.96)	0.677	–	–

### 3.6 Sensitivity analysis

**Figure 9:**
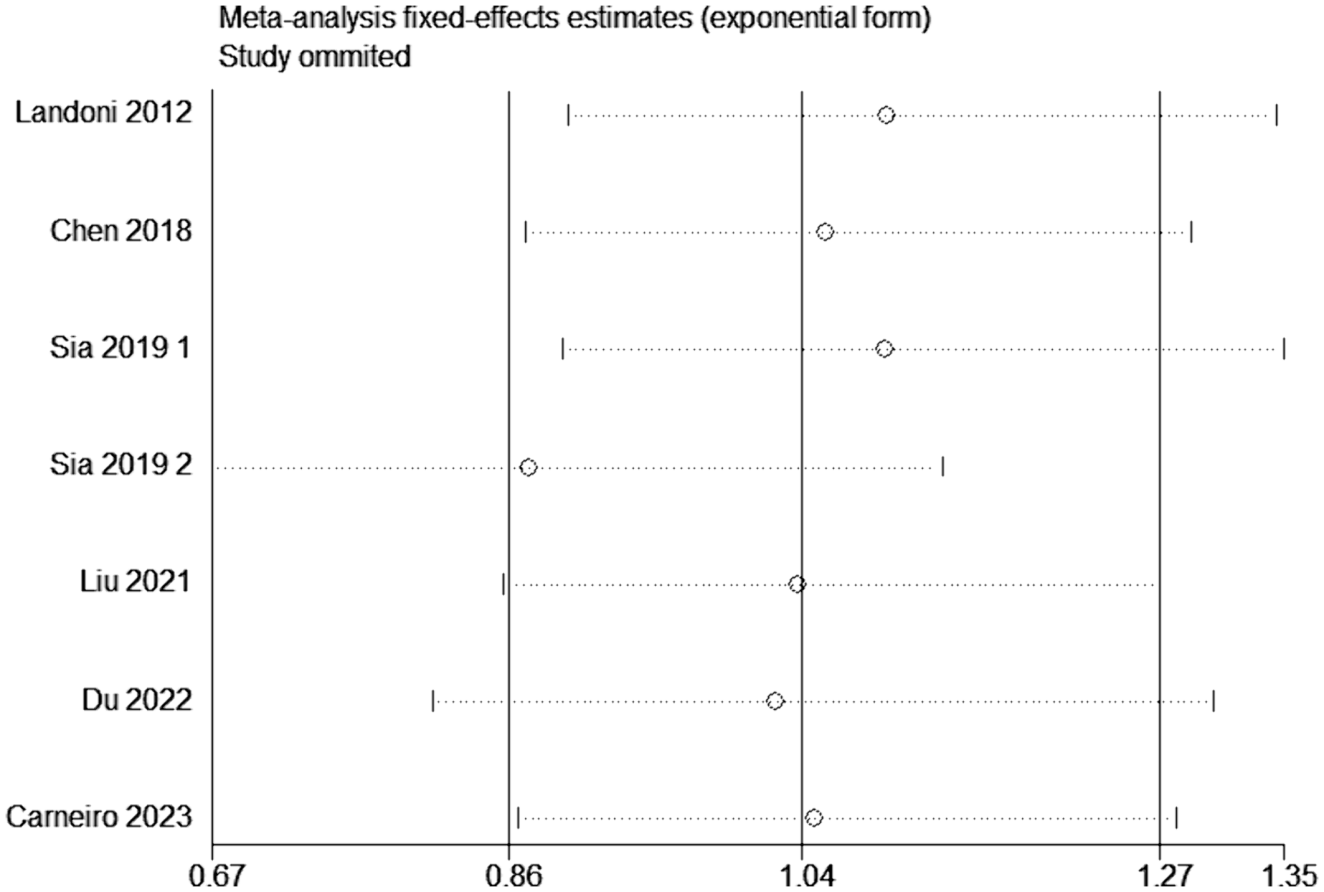
Sensitivity analysis for the meta-analysis (OS).

Sensitivity analysis was conducted by altering the type of effect model or by excluding individual studies from the outcome analysis. In the evaluation of overall survival rates, the systematic removal of individual original studies in a stepwise manner did not elicit substantial alterations in the results, demonstrating consistent findings and minimal fluctuation. This stability suggests that the outcomes obtained from this meta-analysis are resilient and possess a high degree of reliability (Figure 9). In the assessment of heterogeneity, when *I*^2^ exceeds 50%, sensitivity analysis is required. For outcomes with significant heterogeneity, such as the rate of postoperative adjuvant therapy, a sensitivity analysis was conducted by sequentially excluding each included study to assess the stability of the related results. In the investigation concerning the frequency of postoperative adjuvant therapy, the progressive exclusion of individual original studies did not induce substantial changes in the results and exhibited minimal variability, underscoring the robustness and insensitivity of the meta-analysis findings (Supplementary Figure S3).

### 3.7 Publication bias analysis

**Figure 10:**
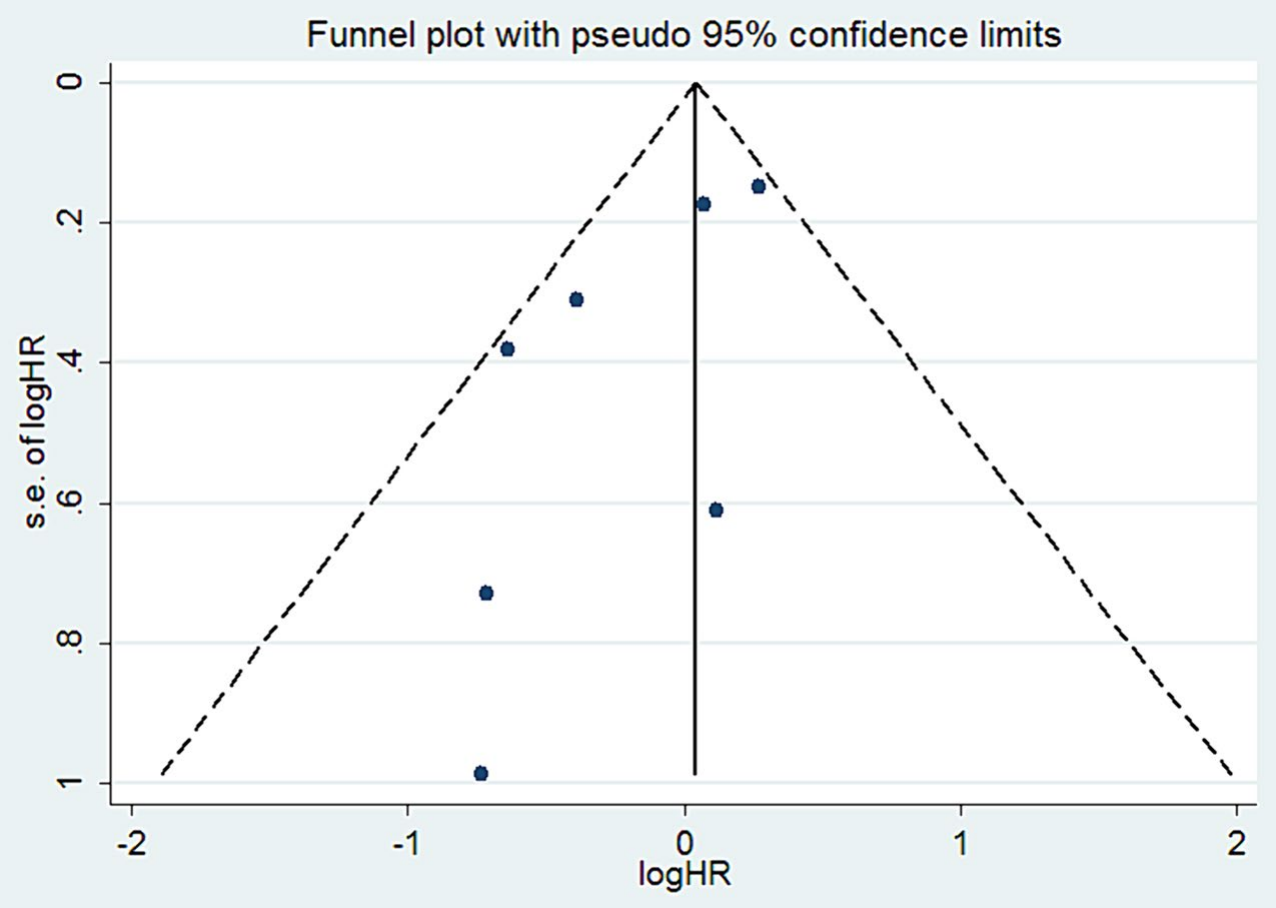
Publication bias detected by funnel plots for OS.

To assess publication bias for the reported OS, we created a funnel plot that demonstrated good symmetry (Figure 10). Additionally, we performed Begg and Egger tests. The publication bias for OS, based on the Begg test, was not significant (*p* = 0.368) (Supplementary Figure S4). Similarly, the Egger test yielded comparable results (*p* = 0.06) (Supplementary Figure S5).

## 4 Discussion

For an extensive period, RH combined with pelvic lymphadenectomy has been the standard surgical approach for patients with cervical cancer at FIGO stages IA2-IB1 (18). Discussions about employing less radical surgical treatments for early-stage cervical cancer patients have been ongoing. Research indicates that in patients who meet specific criteria, less radical surgery can be utilized without compromising survival outcomes, offering a new treatment option for early-stage cervical cancer patients (19, 20).

### 4.1 Key findings of this study

In this study, 3,950 patients in the SH group and 6,271 patients in the RH group were included to assess the efficacy and safety of SH and RH surgeries in the treatment of early-stage cervical cancer patients. Our findings indicate no significant differences between the SH and RH surgery groups in terms of OS, DFS, CCSS, and recurrence rates. However, the mortality rate and the rate of postoperative adjuvant therapy were higher in the SH group than those in the RH group, while the incidence of surgical complications was lower in the SH group. To our knowledge, this study is the first meta-analysis to compare the treatment efficacy of SH and RH in early-stage cervical cancer patients with the largest sample size involved. A total of seven studies reported the number of patient deaths. In the RH group, out of 3,857 patients, 179 died, indicating a mortality rate of 4.64%. In the SH group, out of 2,450 patients, 153 died, suggesting a mortality rate of 6.24%. In the research conducted by Wu et al. (21), it was found that the mortality rate for patients at stage IA2 was 2.7%, while for those at stage IB1, the rate was 7.3%. This disparity in mortality rates highlights the impact of disease progression on patient outcomes. The overall average mortality rate reported in the study by Wu et al. was 5.5%. Also, Wu et al. (21) presented a systematic review on the treatment of early-stage cervical cancer patients who underwent less radical surgery, pooling data from 21 studies involving 2,662 patients. Among these patients, 36.1% were classified as stage FIGO IA1 and 61.0% as IB1. The mortality rate was 4.5% in the RH group and 5.8% in the SH group. The estimated and reported HR values indicate no significant correlation between mortality rates among IA2 stage patients undergoing radical and less radical surgeries, although the mortality rate for IB1 stage disease might increase, which aligns with the findings of our study. Hence, in this present study, to further understand the significant differences in mortality rates between the two groups, a more detailed approach was adopted, conducting subgroup analyses based on tumor staging, LVSI status, LN status, and the type of the original study. The aim of this analysis was to uncover which factors most critically affected the survival outcomes of patients in the SH group. The results indicated that patients who were treated with SH at the positive LVSI status and those in the early stage of IB1 cervical cancer exhibited significantly higher mortality rates compared to those undergoing RH. A study (22) suggests that the positive status of LVSI in early cervical cancer tissue may significantly increase the risk of LN metastasis, thereby seriously affecting the prognosis of patients. For patients with cervical cancer, the relationship between LVSI and clinical prognosis exhibits a complexity not witnessed in the straightforward correlations seen with parametrial infiltration and LN metastasis. The formation of neoangiogenesis and neolymphangiogenesis crucial for tumor expansion predominantly originates from the cervical stroma. Theoretically, an increase in cervical stromal infiltration depth escalates the chances of intravascular spread of cancer emboli. Therefore, a rise in the depth of tumor infiltration corresponds to an augmented frequency of LVSI. For early cervical cancer patients receiving SH treatment, the meticulous preoperative assessment of LVSI, ideally accomplished through methods such as cervical conization, emerges as a pivotal step. This step significantly enhances the condition evaluation process and treatment efficacy, laying a solid foundation for treatment planning. In our analysis, roughly 30.48% of patients in the SH cohort and 20.02% in the RH cohort required postoperative adjuvant therapy. However, given the nature of our systematic review and meta-analysis, the specific rationales behind the postoperative adjuvant therapy in individual studies remain beyond our scope. Previous studies identifying the primary factors leading to postoperative adjuvant therapy (10–17) cited tumor depth stromal invasion, positive LN metastasis, LVSI, positive margins, grade 3 tumors, and parametrial invasion. Particularly noteworthy was the investigation by Wu et al. (21) revealing a higher utilization rate of adjuvant therapy (comprising radiation or chemotherapy) in the SH group at 30.7% compared to 16.7% in the RH group, a trend closely mirrored in our findings. In managing early-stage cervical cancer, the inclination towards less radical surgical approaches aims to mitigate the associated morbidity linked to aggressive surgical interventions. Notably, four studies documented surgical complications, with incidences of 24.4% (51 out of 209 patients) in the RH group and 8.63% (17 out of 197 patients) in the SH group. Predominant complications encompassed lymphedema, lymphocysts, and the occurrence of urinary incontinence. Importantly, for patients in the SH group, the higher the postoperative adjuvant treatment rate, the higher the incidence of treatment-related complications. Therefore, it is crucial to accurately screen patients who are suitable for SH and avoid them receiving adjuvant therapy after surgery.

### 4.2 Discussion on the use of SH in minimally invasive or open surgery for early cervical cancer patients

According to a study by Violante Di Donato et al. (23), the ten-year OS rates of early low-risk cervical cancer patients who underwent minimally invasive RH were not significantly different from those who underwent open RH (98% vs. 96%; *p* = 0.995). However, open RH remains the standard surgical method for cervical cancer patients, and minimally invasive RH should only be performed in clinical trials. In another study by Giacomo Corrado et al. (24), no significant differences were found in recurrence rates, distant metastasis risk, DFS, and OS between minimally invasive and open RH for patients with IB1 to IB2 stage cervical cancer. Nevertheless, the aforementioned studies did not explore the safety of minimally invasive SH for early low-risk cervical cancer patients. The LACC Trial study (25) showed that patients with tumor diameters >2 cm had worse prognoses with minimally invasive surgery, while those <2 cm did not reach statistical significance due to the small sample size. However, based on the number of DFS events, minimally invasive surgery (7/75) still demonstrated a higher occurrence compared to open surgery (0/65). On a related note, Liu et al. (13) examined the impact of minimally invasive surgery on the survival rate of patients with IA2 stage cervical cancer and found no significant change in survival rate. It is important to highlight that this study did not include patients with IB1 stage cancer. In the ConCerv trial (19), 96% of the patients underwent minimally invasive SH surgery, and no recurrences were observed during a 2-year follow-up period. Another trial, the SHAPE trial, demonstrated that neither open nor minimally invasive surgery affected the recurrence risk for early low-risk cervical cancer patients. Overall, for those patients who meet specific criteria, minimally invasive surgery to perform SH appears to be an effective alternative strategy to open surgery. However, this assumption needs to be further verified through more high-quality RCTs.

### 4.3 How to screen suitable patients for SH

In recent years, a growing body of research has been dedicated to exploring the feasibility of employing less radical surgical methods for managing early-stage cervical cancer patients (26–32). The primary objective of employing SH treatment for early-stage cervical cancer patients is to accurately stratify patients based on the presence or absence of parametrial invasion risk prior to surgical intervention. Patients with favorable pathological features are associated with a notably low incidence of parametrial invasion, eliminating the need for complete removal of the parametrial area (33–35). A significant update in the latest NCCN guidelines (36) pertains to the management of low-risk patients with IA2-IB1 stage cervical cancer following cone biopsy. Specifically, patients meeting stringent criteria including the absence of LVSI, negative surgical margins, histologically confirmed squamous carcinoma or ordinary type adenocarcinoma (limited to G1 or G2), a tumor size not exceeding 2 cm, an invasion depth within 10 mm, and lacking radiographic evidence of metastasis, may be candidates for cervical conization and pelvic LN dissection (or sentinel LN evaluation) if fertility preservation is desired. Otherwise, SH + pelvic LN dissection (or sentinel LN evaluation) is recommended. However, the latest European guidelines (37) diverge from the 2023 NCCN version, recommending SLN biopsy for patients at the IA2 stage contingent upon LVSI status, while endorsing radical hysterectomy for those at IB1 stage. Notably, a study by Landoni et al. (14) shows that patients at stages IB1-IIA (tumor diameter ≤ 3 cm) undergoing Piver type I surgery (extrafascial hysterectomy, bilateral salpingectomy, and upper third vaginal resection), exhibit comparable recurrence and survival outcomes. In summary, our study suggests that for cervical cancer patients at stage IA2 and those with negative LVSI, SH treatment is an effective and safe alternative to RH. However, for patients with positive LVSI and those at stage IB1, SH treatment may adversely impact their mortality risk. Although the quality of certain randomized controlled trials reviewed within this investigation is susceptible to moderate bias, limiting the precision of our conclusions, patients presenting with early-stage cervical cancer appear to demonstrate favorable overall survival rates regardless of the surgical approach adopted, particularly those at the IA2 stage devoid of LVSI, reflecting discrepancies with the 2023 NCCN guidelines but echoing the sentiments of the 2023 European guidelines. Moreover, for early-stage (IA2 to IB1) cervical cancer patients, SH significantly reduces complications associated with surgery compared to RH. Efforts to refine patient selection criteria for less extensive surgical interventions hinge upon forthcoming evidence stemming from high-caliber randomized controlled trials.

### 4.4 Ongoing research

The most recent findings from the SHAPE trial (38) were presented at the 2023 American Society of Clinical Oncology annual meeting. Over an average follow-up of 4.5 years, the 3-year pelvic recurrence rate for patients undergoing SH was 2.52%, compared to 2.17% for those undergoing RH. This resulted in a marginal variance of 0.35%, aligning with the upper limit of the 95% confidence interval at 2.32%, which fell below the predefined upper limit of 4%. Furthermore, the prevalence of early postoperative surgical complications within a 4-week window stood at 42.6% in the SH group compared to 50.6% in the RH group (*p* = 0.04). Subsequently, the occurrence of delayed postoperative adverse events following the initial 4 weeks recorded figures of 53.6% in the SH group and 60.5% in the RH group (*p* = 0.08). This study indicates that for early low-risk cervical cancer patients, SH is not inferior to RH. The Gynecologic Oncology Group trial 278 is evaluating the impact of non-radical surgery on functional outcomes such as lymphedema, bowel, and sexual functions in patients with stage IA1 with LVSI and IA2 to IB1 stage (tumor diameter ≤ 2 cm). The publication of these high-level evidence clinical study results may provide strong evidence-based medicine support for the use of less radical surgery in treating early low-risk cervical cancer patients.

### 4.5 Study highlights and limitations

This study represents the latest meta-analysis work in the field concerning this topic. By extensively collecting and synthesizing related literature from multiple databases, this research meticulously selected high-quality RCTs and cohort studies. This is the first time that gold-standard oncological outcomes, such as OS, have been incorporated into a comprehensive evaluation. Furthermore, the study conducted a thorough analysis of key indicators, including DFS, CCSS, mortality rates, recurrence rates, rates of postoperative adjuvant therapy, and rates of surgical complications, leading to more comprehensive and reliable conclusions. In conducting subgroup analyses, this study specifically considered key factors affecting treatment outcomes, including FIGO stages, types of studies, and LVSI status, and LN status. To ensure the fairness and reliability of the research, publication bias was assessed through funnel plots, Egger’s test, and Begg’s test for the included literature, showing no significant bias. Additionally, sensitivity analyses confirmed the robustness of the meta-analysis results. Overall, the findings of this study provide important reference for the individualized surgical choices of patients with early-stage cervical cancer and may have significant implications for clinical practice. However, there are some limitations to this study that need to be acknowledged. (1) There is significant variability in the criteria for selecting SH among original studies. It is important to note that this manuscript does not primarily utilize the Querleu-Morrow classification because Simple Hysterectomy, Querleu-Morrow Type A, and Piver type I hysterectomy represent different surgical approaches. Specifically, there is a distinction in definition between Querleu-Morrow type A and Piver type I. These differences somewhat limit the ability to further precisely analyze the study results. (2) There is a scarcity of research on perioperative and long-term complications associated with SH, which is a key driving factor for considering SH as an alternative to RH. (3) Some key findings are derived from large-scale population-based cohort registries, which have a lower level of evidence compared to RCTs. (4) Based on the available primary literature, it is currently not possible to differentiate recurrence rates into categories of pelvic and extra-pelvic recurrences. In conclusion, while this study offers guidance for clinical practice, further research is required to accurately identify patients who meet these treatment criteria. The limitations of this study are expected to be addressed in the ongoing large-scale prospective studies.

## 5 Conclusion

The meta-analysis conducted in this study elucidates the following key findings: (1) For cervical cancer patients at stage IA2 and those with negative LVSI, no significant differences were found between the SH and RH groups in terms of OS, DFS, CCSS, RR, and mortality, indicating that the type of surgery does not affect the long-term survival outcomes for these patients. (2) For patients at stage IB1 or IA2 with positive LVSI, although no significant differences were observed between the SH and RH groups in OS, DFS, CCSS, and recurrence rate, a notable increase in mortality was observed in the SH group, suggesting that the type of surgery may increase the mortality risk for these patients. (3) In terms of safety, the SH group experienced significantly fewer surgery-related complications compared to the RH group. Significantly, among patients in the SH group, an increase in the rate of postoperative adjuvant treatment is associated with a higher occurrence of treatment-related complications. Therefore, when choosing surgical treatment options for early-stage cervical cancer patients, a comprehensive consideration of the specific characteristics of the case is essential, including the staging of the tumor, LVSI status, the patient’s personal preferences, and the expected progression of the disease, among other factors. Through a thorough assessment and careful weighing of the pros and cons, clinicians can furnish patients with an evidence-based and personalized treatment plan. Such an approach not only maximizes treatment efficacy but also reduces surgery-related complications, thereby benefiting early-stage cervical cancer patients to the greatest extent. Future research will be directed towards how to more accurately select patients suitable for less extensive surgery, which will become a key area of study in this field.

## Data availability statement

The raw data supporting the conclusions of this article will be made available by the authors, without undue reservation.

## Author contributions

SZ: Conceptualization, Data curation, Formal analysis, Investigation, Methodology, Software, Validation, Visualization, Writing – original draft, Writing – review & editing. SX, YX, PY and CH: Conceptualization, Data curation, Investigation, Writing – review & editing. LL and XJ: Funding acquisition, Supervision, Writing – review & editing.

## Supplementary material

### FIGURE S1

Risk of bias graph for randomized controlled trials (RCTs).

### FIGURE S2

Risk of bias summary for randomized controlled trials (RCTs).

### FIGURE S3

Sensitivity analysis for the meta-analysis (Postoperative adjuvant therapy rate).

### FIGURE S4

Publication bias detected by Begg’s funnel plots for OS.

### FIGURE S5

Publication bias detected by Egger’s funnel plots for OS.
