# Supplementary material for: Efficacy and safety analysis of non-radical surgery for early-stage cervical cancer (IA2 ~ IB1): a systematic review and meta-analysis
Source: Front Med (Lausanne). 2024 Apr 30;11:1337752. doi: 10.3389/fmed.2024.1337752 (PMC11091289; doi:10.3389/fmed.2024.1337752)
Supplement: Supplementary file 1 [file Data_Sheet_1.PDF]

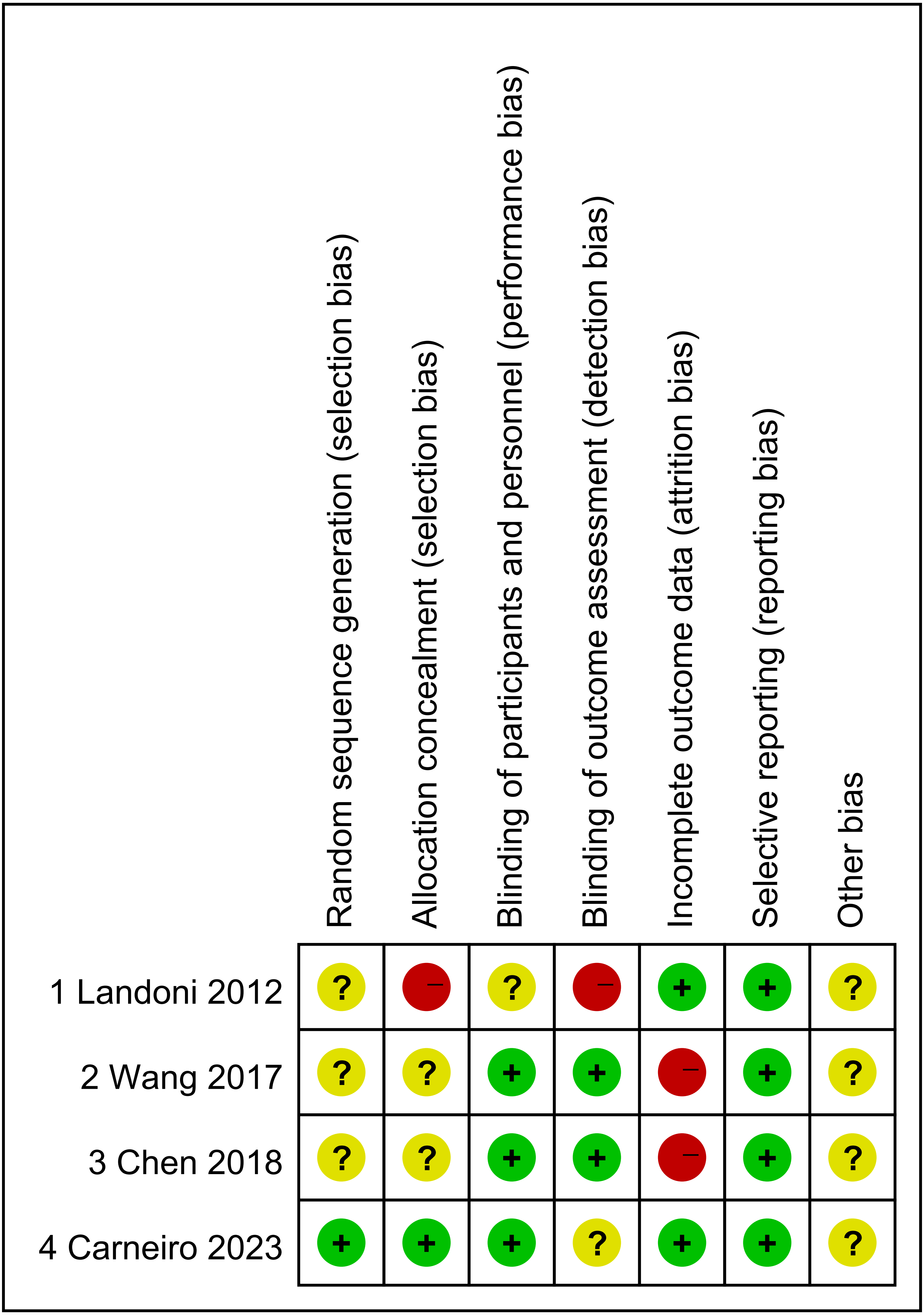
Random sequence generation (selection bias)
Allocation concealment (selection bias)
Blinding of participants and personnel (performance bias)
Blinding of outcome assessment (detection bias)
Incomplete outcome data (attrition bias)
Selective reporting (reporting bias)
Other bias
1 Landoni 2012 ? − ? − + + ?
2 Wang 2017 ? ? + + − + ?
3 Chen 2018 ? ? + + − + ?
4 Carneiro 2023 + + + ? + + ?
