# Supplementary figures and images for: Efficacy and safety analysis of non-radical surgery for early-stage cervical cancer (IA2 ~ IB1): a systematic review and meta-analysis
Source: Front Med (Lausanne). 2024 Apr 30;11:1337752. doi: 10.3389/fmed.2024.1337752 (PMC11091289; doi:10.3389/fmed.2024.1337752)

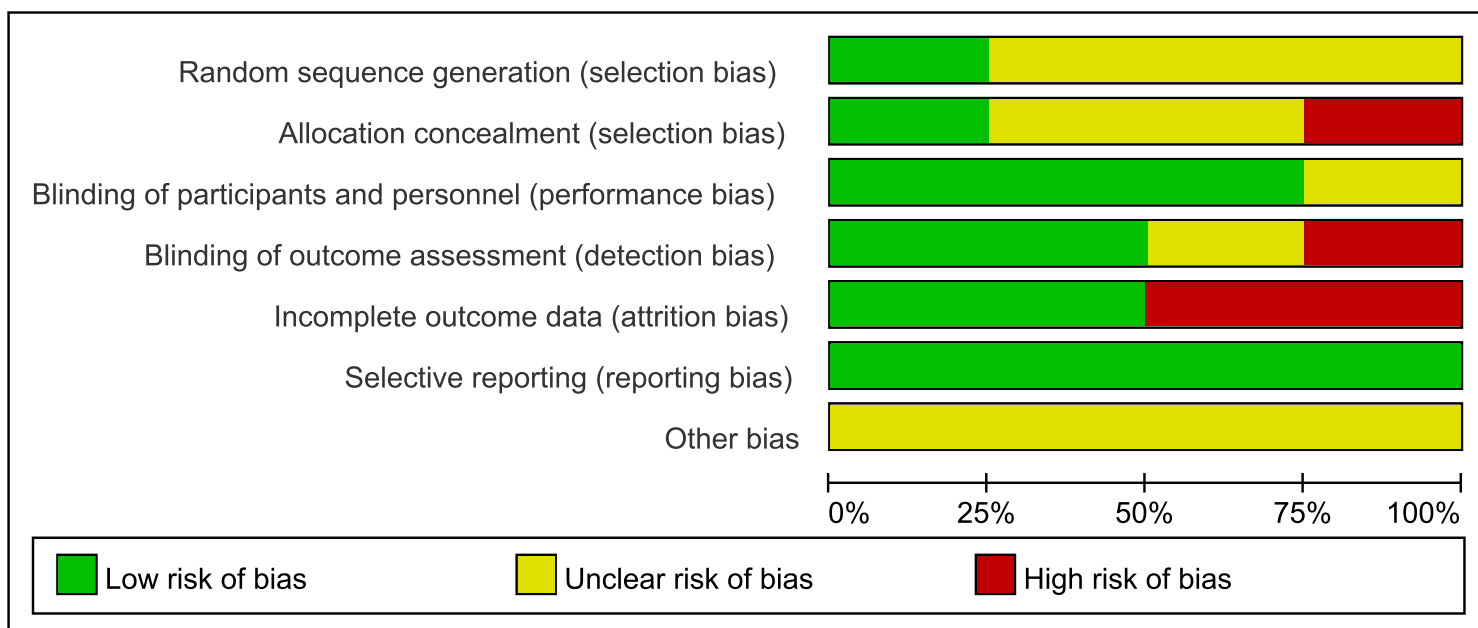

Supplement: Supplementary file 2 [file Data_Sheet_2.PDF]

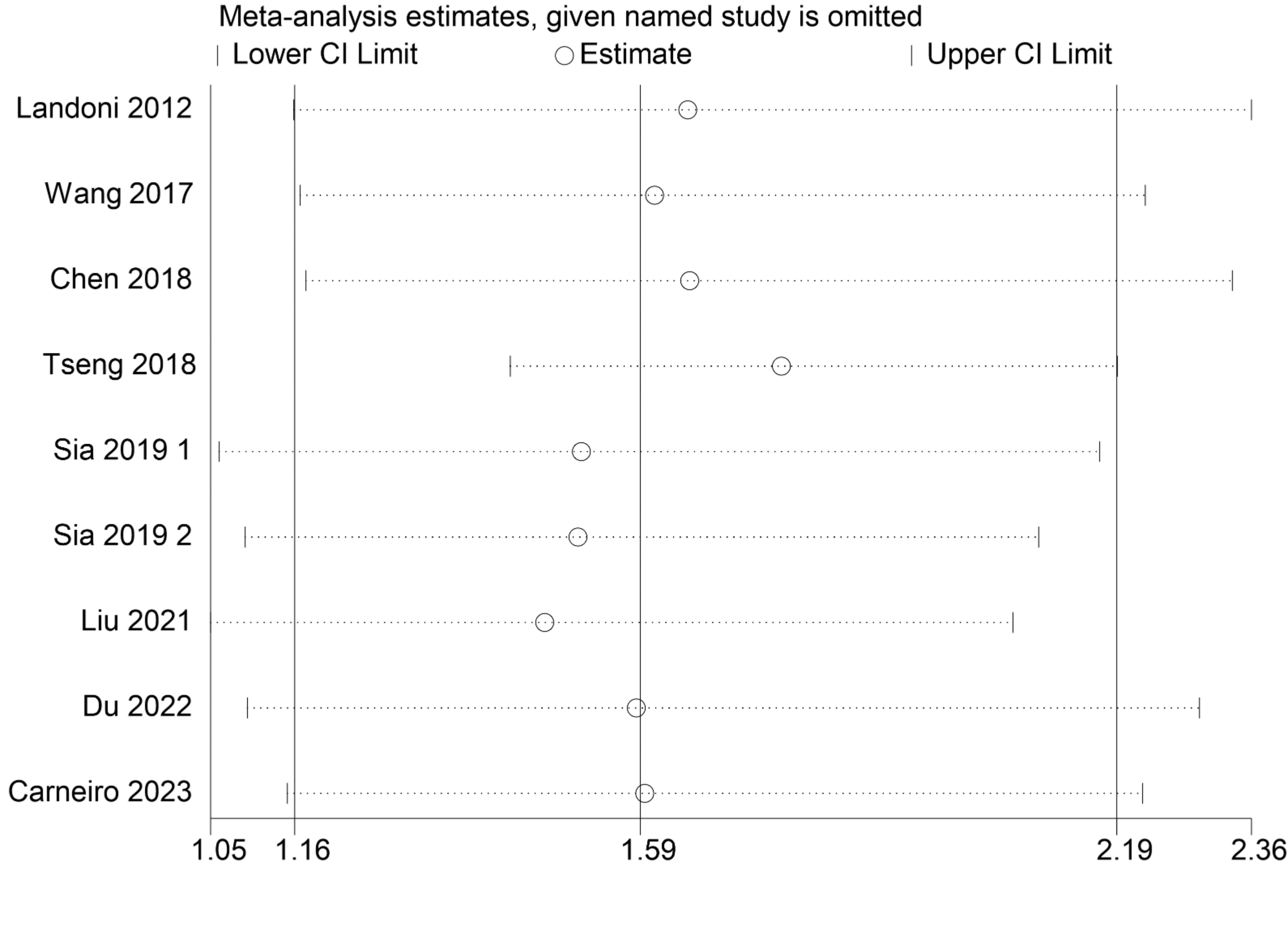

Supplement: Supplementary file 3 [file Image_1.JPEG]

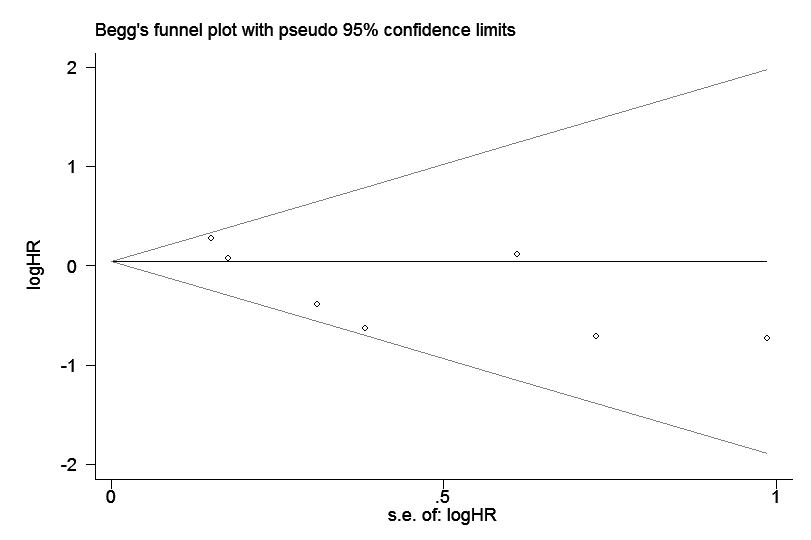

Supplement: Supplementary file 4 [file Image_2.TIF]

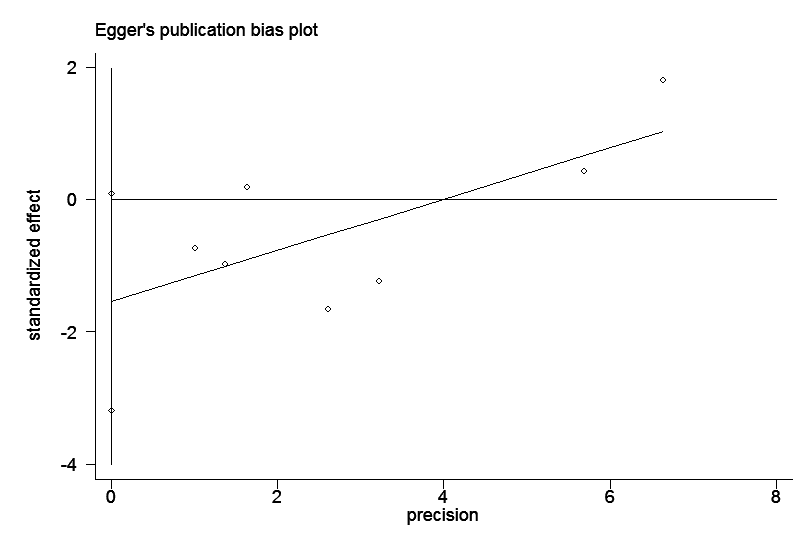

Supplement: Supplementary file 5 [file Image_3.TIF]
