# Supplementary material for: Efficacy and safety analysis of non-radical surgery for early-stage cervical cancer (IA2 ~ IB1): a systematic review and meta-analysis
Source: Front Med (Lausanne). 2024 Apr 30;11:1337752. doi: 10.3389/fmed.2024.1337752 (PMC11091289; doi:10.3389/fmed.2024.1337752)
Supplement: Supplementary file 6 [file Table_1.docx]

| Studies | Selection | | | | Comparability | Outcome | | | Scores |
| --- | --- | --- | --- | --- | --- | --- | --- | --- | --- |
|  | Representativeness of the exposed cohort | Selection of the non-exposed cohort | Ascertainment of exposure | Outcome of interest not present at start of study | Comparability of cohorts on the basis of the design or analysis | Assessment of outcome | Follow-up long enough for outcomes to occur | Adequacy of follow up of cohorts |  |
| Tseng 2018 | ★ | ★ | ★ | ★ | ★★ | ★ | ★ | - | 8 |
| Sia 2019 1 | ★ | ★ | ★ | ★ | ★★ | ★ | ★ | - | 8 |
| Sia 2019 2 | ★ | ★ | ★ | ★ | ★★ | ★ | ★ | - | 8 |
| Liu 2021 | ★ | ★ | ★ | ★ | ★★ | ★ | ★ | - | 8 |
| Du 2022 | ★ | ★ | ★ | ★ | ★★ | ★ | ★ | - | 8 |

Supplement Table 1. Literature Quality Evaluation Form (NOS)
